# Abnormal dopaminergic modulation of striato-cortical networks underlies levodopa-induced dyskinesias in humans

**DOI:** 10.1093/brain/awv096

**Published:** 2015-04-15

**Authors:** Damian M. Herz, Brian N. Haagensen, Mark S. Christensen, Kristoffer H. Madsen, James B. Rowe, Annemette Løkkegaard, Hartwig R. Siebner

**Affiliations:** 1 Danish Research Centre for Magnetic Resonance, Centre for Functional and Diagnostic Imaging and Research, Copenhagen University Hospital Hvidovre, Hvidovre, Denmark; 2 Department of Neurology, Copenhagen University Hospital Bispebjerg, Copenhagen, Denmark; 3 Department of Nutrition, Exercise and Sports, University of Copenhagen, Copenhagen, Denmark; 4 Department of Neuroscience and Pharmacology, University of Copenhagen, Copenhagen, Denmark; 5 DTU Compute, Technical University of Denmark, Lyngby, Denmark; 6 Department of Clinical Neurosciences, Cambridge University, Cambridge, UK; 7 Medical Research Council Cognition and Brain Sciences Unit, Cambridge, UK; 8 Behavioural and Clinical Neuroscience Institute, Cambridge, UK; 9 Department of Clinical Medicine, Faculty of Health and Medical Sciences, University of Copenhagen, Copenhagen, Denmark

**Keywords:** Parkinson's disease, levodopa-induced dyskinesia, connectivity, functional MRI, dynamic causal modelling

## Abstract

Dopaminergic signalling in the striatum contributes to reinforcement of actions and motivational enhancement of motor vigour. Parkinson's disease leads to progressive dopaminergic denervation of the striatum, impairing the function of cortico-basal ganglia networks. While levodopa therapy alleviates basal ganglia dysfunction in Parkinson's disease, it often elicits involuntary movements, referred to as levodopa-induced peak-of-dose dyskinesias. Here, we used a novel pharmacodynamic neuroimaging approach to identify the changes in cortico-basal ganglia connectivity that herald the emergence of levodopa-induced dyskinesias. Twenty-six patients with Parkinson's disease (age range: 51–84 years; 11 females) received a single dose of levodopa and then performed a task in which they had to produce or suppress a movement in response to visual cues. Task-related activity was continuously mapped with functional magnetic resonance imaging. Dynamic causal modelling was applied to assess levodopa-induced modulation of effective connectivity between the pre-supplementary motor area, primary motor cortex and putamen when patients suppressed a motor response. Bayesian model selection revealed that patients who later developed levodopa-induced dyskinesias, but not patients without dyskinesias, showed a linear increase in connectivity between the putamen and primary motor cortex after levodopa intake during movement suppression. Individual dyskinesia severity was predicted by levodopa-induced modulation of striato-cortical feedback connections from putamen to the pre-supplementary motor area (*P*_*corrected*_ = 0.020) and primary motor cortex (*P*_*corrected*_ = 0.044), but not feed-forward connections from the cortex to the putamen. Our results identify for the first time, aberrant dopaminergic modulation of striatal-cortical connectivity as a neural signature of levodopa-induced dyskinesias in humans. We argue that excessive striato-cortical connectivity in response to levodopa produces an aberrant reinforcement signal producing an abnormal motor drive that ultimately triggers involuntary movements.

## Abstract (precis)

Abnormal dopaminergic modulation of connectivity between the putamen and cortex is thought to underlie the emergence of levodopa-induced dyskinesias. Herz *et al*. confirm this directly by showing that in individuals with Parkinson's disease who have taken a single dose of levodopa, changes in connectivity preceding dyskinesias accurately predict their severity.

## Graphical abstract

**Figure:**
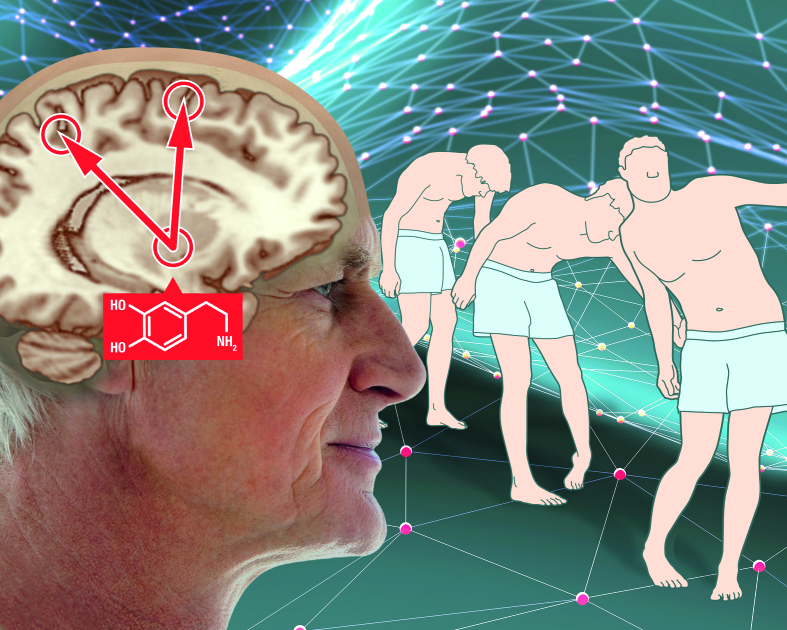
Abnormal dopaminergic modulation of connectivity between the putamen and cortex is thought to underlie the emergence of levodopa-induced dyskinesias. Herz *et al*. confirm this directly by showing that in individuals with Parkinson's disease who have taken a single dose of levodopa, changes in connectivity preceding dyskinesias accurately predict their severity.

## Introduction

The neurotransmitter dopamine provides a reinforcement signal in the striatum that facilitates appropriate actions and suppresses inappropriate actions (Redgrave *et al*., 2011). Neurobiologically, this is implemented by inducing long-lasting plasticity in specific cortico-striatal synapses, which results in privileged processing of the reinforced cortico-striatal inputs in the cortico-basal ganglia-thalamo cortical re-entry loops (Calabresi *et al*., 2007). This decreases the ‘threshold’ to generate the specific action in the future and thus prioritizes competing action programs according to previous action outcomes and current goals (Redgrave *et al*., 2011). Nigro-striatal dopaminergic signalling is also thought to enhance motor vigour and to establish motor routines and habits (Robbins and Everitt, 2007; Graybiel, 2008).

In Parkinson's disease, progressive degeneration of dopaminergic midbrain neurons results in functional de-afferentation of the putamen, the main motor input region of the basal ganglia (Lang and Lozano, 1998*a*, *b*). The dopamine precursor levodopa, usually in combination with dopamine agonists, remains the standard treatment for alleviation of motor symptoms in Parkinson's disease (Hauser, 2009). However, depending on the dose of levodopa and duration of the disease (Cilia *et al*., 2014), many patients with Parkinson's disease start to experience disabling motor fluctuations, including involuntary ‘dyskinesia’ movements (Ahlskog and Muenter, 2001; Van Gerpen *et al*., 2006). These levodopa-induced dyskinesias (LID) occur most frequently when the dopamine effect reaches its peak (i.e. peak-of-dose dyskinesia).

Animal models of Parkinson's disease have shown that the non-physiological dopaminergic stimulation of the putamen induces maladaptive plastic changes in the cortico-basal ganglia motor loops, resulting in an excessive striato-cortical drive during high levels of striatal dopamine (Cenci and Lundblad, 2006). This excessive network activity is, however, not contingent on previous action outcomes as in the healthy brain. Instead, activity largely depends on the dynamics of levodopa-induced changes in striatal dopamine and the degree of nigro-striatal neurodegeneration because the de-afferentated putamen is no longer able to store and release dopamine in a controlled manner (Cenci and Lundblad, 2006). The prevailing notion is that abnormal dopaminergic modulation of the cortico-basal ganglia motor loops underlies the emergence of LID (Obeso *et al*., 2000; Cenci and Lundblad, 2006; Calabresi *et al*., 2007; Ulusoy *et al*., 2010). However, this claim has never been tested in humans, in part because the movement artefacts caused by LID severely impairs the quality of brain imaging data.

**Figure 1:**
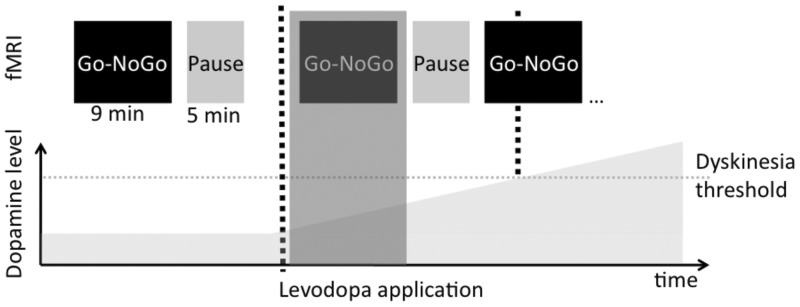
**Study design**. All patients were in a practical ‘OFF-state’ during the initial scans (OFF session). In each session, the motor task was followed by a 5 min pause to avoid fatigue. After the OFF scan, patients received 200 mg of fast-acting soluble levodopa (Madopar Quick®, La Roche) and the MRI scan was continued immediately with the same order of scans as in the OFF (post-levodopa Scan 1 – pause – post-levodopa Scan 2). The time elapsing between application of levodopa and initiation of the functional post-levodopa scans was ∼15 min and did not differ between groups. A medical doctor (D.M.H.) was continuously present inside the scanner room during MRI acquisition to visually observe whether LID emerged after levodopa intake. Data acquisition was discontinued as soon as patients developed LID. This enabled us to capture the progressively emerging neural response to levodopa in patients with Parkinson's disease without the problem of dyskinesia-related movement artefacts. For patients that did not develop apparent LID inside the scanner MRI recordings were stopped after two post-levodopa sessions (i.e. ∼45 min after levodopa intake). We assessed the dopaminergic modulation of neural network connectivity only in the scan immediately following levodopa intake (post-levodopa Scan 1, see shaded area), because four patients already developed dyskinesias in the second scan after levodopa intake (post-levodopa Scan 2). fMRI = functional MRI.

To address this issue, we used a novel functional MRI design to investigate acute changes in neural connectivity between motor areas in the striatum and cortex in response to a single dose of levodopa. In patients with Parkinson's disease with and without peak-of-dose LID, dynamic changes in striato-cortical connectivity were assessed with whole-brain functional MRI, while patients performed a simple motor task. The task required patients to press a button with their right or left index finger or to refrain from any response (referred to as NoGo) contingent on three pre-learned arbitrary visual cues. A first functional MRI run was acquired after prolonged withdrawal of dopaminergic medication had induced a dopamine-depleted state (‘OFF’). The functional MRI measurements continued immediately following the intake of 200 mg fast-acting soluble levodopa (‘post-levodopa’) before any dyskinetic movements emerged (Fig. 1). A conventional voxel-wise comparison revealed that in patients with Parkinson's disease who would later develop LID, levodopa triggered excessive No-Go activity in the pre-supplementary motor area (preSMA) and the bilateral putamen in the pre-dyskinesia period (Herz *et al*., 2014b).

This finding prompted us to apply a model-based directional connectivity analysis, called dynamic causal modelling (DCM) (Friston *et al*., 2003), to assess the acute changes in network connectivity within the cortico-basal ganglia loops after levodopa intake. Focusing on the time period preceding LID (i.e. the post-levodopa Scan 1) and the task context of response inhibition (NoGo), our pharmacodynamic functional MRI approach allowed for the first time to trace the emergence of abnormal connectivity patterns preceding LID. We predicted that patients who would later develop peak-of-dose LID would express an abnormal enhancement of connectivity in networks connecting the putamen and cortex in response to dopamine.

## Patients and methods

We included 13 Parkinson’s disease patients with clinically diagnosed peak-of-dose choreiform dyskinesias (LID group), and 13 Parkinson’s disease patients without dyskinesias (No-LID group). None of the patients had biphasic dyskinesias. Exclusion criteria were tremor, dementia or major psychiatric illness, implanted pacemakers or other general contra-indication regarding magnetic resonance, and sedatives or serotonergic medication. In accordance with the Declaration of Helsinki, all participants gave their informed consent to the study, which was approved by the local ethics committee (study-nr: H-2-2010-146).

Dyskinesias were assessed using the Unified Dyskinesia Rating Scale. Both groups were matched with regard to age, sex, handedness (Edinburgh Handedness Inventory), education, cognitive function (Mini-Mental Status Examination and Montreal Cognitive Assessment), and impulsive personality trait (Barratt Impulsiveness Scale, BIS-11). Patients with LID received a significantly higher levodopa-equivalent daily dose (LEDD) compared to the No-LID group. This difference was driven by differences in the daily dose of levodopa, while there were no differences in the doses of dopamine agonists (Supplementary Table 1). However, LID and No-LID patients did not differ in disease duration, disease severity [Unified Parkinsons Disease Rating Scale-III (UPDRS-III), OFF medication] or clinical response to levodopa (UPDRS-III ON versus UPDRS-III OFF medication). Additionally, there was no difference with regard to the number of patients receiving dopamine agonists next to levodopa therapy. All details and group comparisons of clinical specifications can be found in Supplementary Table 1.

### Study design

We acquired whole-brain functional MRI scans in the ‘practical OFF medication state’ after prolonged withdrawal of all dopaminergic medication and continuously after patients had received 200 mg soluble levodopa + 50 mg benserazide (Madopar Quick®, La Roche). Dopaminergic medication was withdrawn according to six half-lives of the respective medication and at least 12 h. In case dopamine agonists had to be stopped several days before the experiment, we simultaneously increased levodopa dosage to reach a similar level of LEDD. We chose this relatively rigorous washout phase, because the response to levodopa is more pronounced after a washout phase compared to standard treatment (Nutt *et al*., 1994; Zappia *et al*., 1997). Given the long duration response to levodopa, which builds up and decreases slowly—at least in earlier stages of the disease (Nutt and Holford, 1996; Hauser and Holford, 2002)—even our rigorous approach did not lead to a complete depletion of the effects of all extrinsic dopamine. Nevertheless, we maintain that our approach applied was sufficient given the purpose of the study. There were no differences in disease severity (UPDRS-OFF) between groups after the washout phase, the clinical response to 200 mg levodopa (UPDRS ON versus OFF) was similar in the two groups and robustly induced dyskinesias in the LID group, but none in the No-LID group. The time line of functional MRI measurements is illustrated in Fig. 1. This novel pharmacodynamic functional MRI design allowed us to map the gradual effect of increasing dopamine levels on connectivity in cortico-basal ganglia motor loops before the onset of LID rather than contrasting an ‘ON medication (dyskinetic)’ with an ‘OFF medication (non-dyskinetic) state’. Because four patients developed LID already during the second scan after levodopa intake (post-levodopa Scan 2), we only used the first scan after levodopa intake (post-levodopa Scan 1) for assessing the gradual network response to dopamine in patients with and without the subsequent onset of LID. Mean time from application of levodopa to commencement of functional MRI scans was not different between groups {post-levodopa Scan 1: LID 13 ± 3 min [mean ± standard deviation (SD)] versus No-LID 15 ± 5 min, *P* = 0.337; post-levodopa Scan 2: LID 32 ± 3 min versus No-LID 33 ± 6 min, *P* = 0.375; independent samples *t*-tests}.

The event-related functional MRI design used a stimulus-response mapping task, in which participants pressed a mouse button with their right index finger (Right), left index finger (Left), or refrained from any motor response (NoGo) depending on the shape of the stimulus (triangle, circle, square). Stimulus-response associations were counterbalanced across participants and groups. Stimuli were presented for 750 ms followed by a central fixation cross (jittered between 2250–3250 ms). The mean intertrial interval was 3500 ms. We used PsychoPy (Peirce, 2007) for stimulus presentation and assigned equal probability to each stimulus. Each session comprised 50 Left, 50 Right and 50 NoGo trials and lasted ∼9 min. All patients performed a training session for ∼5 min before commencement of MRI scans.

### Behavioural analysis

We used a 2 × 3 × 2 analysis of variance (ANOVA) with the factors Group, Run and Task laterality (Left, Right) for analysis of reaction times and the non-parametric Kruskal-Wallis test for analysis of accuracy rates to compare groups in the three conditions (Left, Right, NoGo) and sessions. The mean ± SD is reported for all results, which were thresholded at *P* < 0.05.

### MRI

All MRI data were acquired on a 3-T Verio scanner (Siemens) with a 32-channel head coil. Before functional MRI, we recorded a T_1_-weighted anatomical image (MPRAGE, field-of-view 230 mm, slice thickness 0.9 mm, repetition time 1900 ms, echo time 2.32 ms, flip angle 9° sagittal orientation). The image was co-registered to the functional MRI images, segmented and normalized to the Montreal Neurological Institute (MNI) template. The structural images were used to relate activations observed in the functional MRI analysis to anatomical brain structures of each individual patient.

We acquired 289 functional T_2_*-echo planar images (EPI; field-of-view 192 mm, slice thickness 3.5 mm, slice spacing 0.2 mm, repetition time 1850 ms, echo time 26 ms, flip angle 75°, 36 slices, ascending slice acquisition order, whole brain coverage) in each session. Preprocessing and analysis of MRI data were carried out using Statistical Parametric Mapping (SPM8, Revision update number 4667, Wellcome Trust Centre for Neuroimaging, London, UK) implemented in MATLAB 7.10.2 (Mathworks). The first three volumes were discarded to allow for T_1_-equilibrium effects. The remaining images were realigned to the mean EPI-image of the time-series to correct for small head movements. The resulting images were normalized to a standard EPI template based on the MNI reference brain, resampled to 2 × 2 × 2 mm voxels, and smoothed with an isotropic 8 mm full-width half-maximum Gaussian kernel, to allow for intersubject anatomical differences and allow valid statistical inference according to Gaussian random field theory. Additionally, the data were filtered in the temporal domain using a high-pass filter with a frequency of 1/128 Hz to correct for baseline drifts. We rigidly controlled the functional MRI data for head movements by visual inspection. Additionally, we computed the number of images that were moved >1 mm in relation to the previous image. This affected only 0.17% of all images, confirming that participants did not develop involuntary head movements during the scan, e.g. induced by tremor or dyskinesias.

The statistical analysis of functional MRI data was performed using the general linear model. The design matrix was generated with the canonical haemodynamic response function. Regressors of interest comprised Right, Left and NoGo along with their first-order (linear) time-modulation, since we expected a linear increase in neural activity after levodopa intake. Twenty-four regressors were computed based on an expansion of the movement parameters estimated during realignment and included as nuisance covariates in the design matrix to remove residual movements artefacts with spin history effects. Additionally, we included recordings of respiration and cardiac pulsation as nuisance covariates (Lund *et al*., 2006). Controlling for physiological noise is particularly important in pharmacological functional MRI studies, since analysis of neural activity is sensitive to drug-induced changes in physiological parameters (Khalili-Mahani *et al*., 2013). In the current study there were no overall differences between groups (LID, No-LID) or sessions (OFF, ON1, ON2) in pulse rate [mean: 74/min ± 12 (SD); Effect of Session: *P* = 0.314; Effect of Group: *P* = 0.44; Interaction: *P* = 0.155, ANOVA] or respiration frequency (mean: 16/min ± 4; Effect of Session: *P* = 0.072; Effect of Group: *P* = 0.58; Interaction: *P* = 0.588, ANOVA].

Prior to conducting connectivity analyses, we analysed abnormal dopamine-induced changes in neural activity as a functional localizer for the regions of interest of the connectivity analyses. To this end, we compared task-related activity reflecting the linear time-modulation, i.e. the linear changes over time, of right button presses (Right), left button presses (Left) and NoGo in the post-levodopa Scan 1 between the LID and No-LID group using independent samples *t*-tests. The results of these analyses identified the preSMA (centred at *x, y, z*: −4, 8, 58; MNI coordinates) and the bilateral putamen (*x, y, z*: −28 8 −6 and 34 0 4, respectively) as the neural regions, which expressed an enhanced linear increase in activity in LID compared to No-LID patients. This difference was only observed during NoGo, while there were no differences during Left and Right. Thus, analysis of task-related activity identified preSMA and bilateral putamen as the brain regions and the NoGo condition as the context, in which the LID and No-LID group showed differential modulation by dopamine. Furthermore, analysis of main effects of Left, Right and NoGo guided the specifications of DCM parameters (see below). The results related to this functional localizer along with further analyses of neural activity are reported in detail in a companion paper (Herz *et al*., 2014*b*).

### Dynamic causal modelling

DCM was conducted to assess causality and directionality of neural networks and their dynamic modulation by dopamine in patients with and without LID (Friston *et al*., 2003). DCM examines the instantaneous rates of changes in neural activity in response to inputs (an experimental perturbation such as right button presses or activity in another area) underlying the observed changes in the blood-oxygen level-dependent signal using a set of differential equations, comprising an A-matrix (baseline-coupling between regions, which is constant during the task), a B-matrix (modulation of coupling between regions by an external input; here linear changes during NoGo), and a C-matrix (direct inputs to regions; here the different conditions: Left, Right and NoGo) as well as a neurovascular forward model. The neurovascular or haemodynamic forward model assumes a physiologically verified increase in vasodilation and blood flow in the activated region resulting in a change in blood volume and deoxyhaemoglobin content causing the observed changes in the blood-oxygen level-dependent signal. It is constrained to neurovascular physiology by empirical priors and accommodates variations in the haemodynamic response function by optimizing parameters for each subject and modelled region (Daunizeau *et al*., 2011). Based on the results from the functional localizer (Herz *et al*., 2014*b*), we constructed a parsimonious model comprising preSMA and bilateral putamen, as well as bilateral M1 as the region responsible for movement execution (Picard and Strick, 2001). The functional MRI time series were extracted from the respective regions 5 mm in radius from each subject using the first eigenvariate. The coordinates of the regions were based on the functional MRI results of neural activity (Herz *et al*., 2014*b*) (Supplementary Table 2). For each individual participant the respective contrast maps were thresholded at *P*_*uncorrected*_ = 0.05 and the coordinates were moved to the activation maxima closest to the group maximum. To ensure that the new coordinates were localized in the correct anatomical region, activation maxima were overlaid on the individual co-registered structural images and visually controlled. The resulting mean coordinates are shown in Supplementary Table 2. In the DCM, all considered regions shared reciprocal connections except preSMA and M1 (A-matrix) (Picard and Strick, 2001). We considered all events in the model. The direct input (C-matrix) to the regions was defined as the onset of Left, Right and NoGo. These entered the network via the regions, which were activated during the respective conditions (i.e. main effects of Left, Right and NoGo in the analysis of neural activity (Herz *et al*., 2014*b*). Thus, the ‘Left’ cue entered the network via right M1, the ‘Right’ cue entered the network via left M1, and the ‘NoGo’ cue entered the network via preSMA and bilateral putamen. The driving inputs to both motor cortices are likely to be mediated by unmodelled regions, such as premotor areas, but enter the modelled network via M1. The modulatory input (B-matrix) was defined by the linear time-modulation of NoGo-trials during post-levodopa Scan 1. The latter parameter thus served as a surrogate regressor for the effect of levodopa during NoGo-trials.

**Figure 2:**
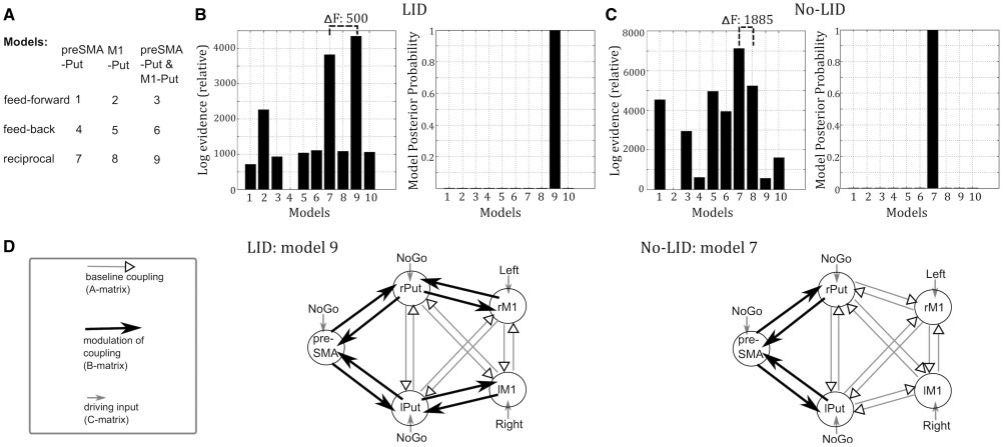
**Model-based connectivity analysis**. (**A**) Models that were constructed and compared within the DCM framework. We also constructed a null model (Model 10) postulating that no connections were modulated by dopamine (not shown). (**B**) Results of Bayesian model selection in the LID group. Model 9 had a far higher likelihood than any other model considered resulting in a posterior probability of >99%. Model 9 explained on average 14.5% variance in the LID group. (**C**) Result of Bayesian model selection in the No-LID group. Here, Model 7 was far superior than any other models considered with a posterior probability of >99%. Model 7 explained on average 12.9% variance in the No-LID group. (**D**) Best network models of the LID (*left*) and No-LID (*right*) group. Driving inputs entered the network via the regions, which were activated during the respective conditions (*right*: left M1; *left*: right M1; NoGo: preSMA, left putamen, right putamen). Note that driving inputs to both motor cortices are likely to be mediated by unmodelled regions, such as premotor areas, but enter the modelled network via M1. Connectivity, which showed a linear time modulation during NoGo-trials (B-matrix), is indicated by black arrows, while unmodulated (baseline) coupling (A-matrix) is indicated by grey arrows.

We created a set of network models to assess which cortico-striatal connections were modulated by levodopa. This analysis step was not exploratory, but tested specific *a priori* defined hypotheses based on the results of changes in neural activity underlying development of LID (Herz *et al*., 2014*b*). Therefore we did not specify all potential models, but specified models sufficient to test our key hypotheses with a systematic exploration of neighbouring model space. The models differed with regards to whether (i) levodopa modulated feedforward connections from the cortex to the putamen, feedback connections from the putamen to the cortex or both (reciprocal); and (ii) connections between putamen and preSMA, between putamen and M1 or between putamen and both preSMA and M1 were modulated. Together with a null-model postulating that no connections were modulated by levodopa, this resulted in 10 models that were constructed (Fig. 2A). The models were then estimated for each patient using an expectation maximization algorithm and compared using Bayesian model selection for fixed effects. The fixed effects analysis assumes that the model structure is the same for each subject (within a group), but the modulation of the coupling can show intersubject variations. Bayesian model selection compares the different models with regard to their likelihood of explaining the data, taking into account model complexity (for details see Penny *et al*., 2004). We can evaluate whether the best model significantly outperforms the other considered models by calculating the difference in relative log-evidence/free energy. A difference in relative log evidence/free energy of > 5 is considered ‘very strong evidence’ and corresponds to a posterior probability of >99% that the winning model generated the data given the model space (Raftery, 1995; Penny *et al*., 2004). Prior to conducting Bayesian model selection we ensured that no outliers were present using Grubbs test. For completeness, we also conducted Bayesian model selection for random effects (Stephan *et al*., 2009). The best model of the LID group was then used to extract connectivity values from each patient for regression analysis.

### Prediction of severity of dyskinesias

Finally, to test whether dopaminergic modulation of connectivity predicted the severity of emerging LID, we applied linear regression analysis. To specifically assess predictions related to dopaminergic modulation of neural connectivity (in contrast to changes in neural activity), we entered the connectivity values of the DCM reflecting levodopa-modulation of connectivity (B-matrix) into a hierarchical multiple linear regression model, in which the coupling values were added into a regression model containing modulation of neural activity (preSMA and putamen, based on the functional localizer). We entered each of the connectivity values reflecting dopaminergic modulation of striato-cortical connectivity (putamen-to-preSMA, putamen-to-M1, preSMA-to-putamen, M1-to-putamen) into the regression model starting with the variable explaining most variance (Scheller *et al*., 2013; Herz *et al*., 2014*a*). In the next step, the variable explaining second most variance was added, etc. and the improvement of predictions of LID assessed in each step by computing the changes in R-square. Since LID always firstly emerged on the side that was most affected by Parkinson's disease, we extracted coupling values of the most affected hemisphere of each individual patient. The results were thresholded at *P* = 0.05. To correct for the extra variables entered into the model we applied the Bonferroni method (Mundfrom *et al*., 2006).

## Results

Patients reported no problems to perform the task. Patients showed high accuracy rates of 96% or more, which were not modulated by group, task, or session (Kruskal-Wallis tests: all *P*_uncorrected_ > 0.1). Patients with peak-of-dose dyskinesias (LID group) and patients without a history of dyskinesias (No-LID group) showed comparable reaction times for visually cued button presses (No-LID: 684 ± 92 ms; mean ± SD; LID 673 ± 103 ms). This was confirmed by analysis of variance (ANOVA), which revealed no main effects of group (*P* = 0.432), session (*P* = 0.286), or task (*P* = 0.356) on mean reaction time and no significant interactions. These findings indicate that differences in network connectivity were not driven by differences in task performance.

After intake of 200 mg levodopa, 10 of 13 patients of the LID group developed dyskinesias while lying inside the scanner (i.e. during the functional MRI experiment). In the remaining three patients, dyskinesias emerged after they had left the scanner room (i.e. after the end of the functional MRI experiment). Four LID patients developed dyskinesias already after post-levodopa Scan 1, i.e. ∼30 min after levodopa intake. Therefore, only the first post-levodopa scan was used for assessing the gradual network response to dopamine (Fig. 1).

To explore the modulation of cortico-striatal connectivity, we applied model-based directional connectivity analysis. This DCM method (Friston *et al*., 2003) has been shown to be reliable in older adults and sensitive to the effects of Parkinson's disease (Rowe *et al*., 2010; Michely *et al*., 2015) giving directional cortico-subcortical connectivity parameters for the motor system that correlate with both cortico-subcortical structural connectivity and behaviour (Herz *et al*., 2014*a*; Rae *et al*., 2015). We applied Bayesian model selection for fixed effects (Penny *et al*., 2004) to compare several network models, which differed with regard to the connections that were modulated by dopamine (Fig. 2A). We found a clear difference between the most likely models in the LID and No-LID groups. The network model postulating dopaminergic modulation of reciprocal connectivity between the putamen and preSMA as well as the putamen and M1 (Model 9) emerged as the most likely model in patients who later developed LID with a posterior probability of >99% (difference in free energy of Model 9 compared to the next best model: 500). In contrast, for patients who did not develop LID, the model postulating modulation of reciprocal connectivity between the putamen and preSMA, but not M1 (Model 7) exceeded the probability of all other models with a posterior probability of >99% (difference in free energy of Model 7 compared to the next best model: 1885). The DCM results are illustrated in Fig. 2B–D (see also Supplementary Table 3 for estimated parameters of the respective models). Bayesian model selection for random effects produced consistent results, namely the highest exceedance probability (i.e. the probability of a given model being more likely than any other model considered) for Model 9 in the LID group (0.43) and Model 7 in the No-LID group (0.34).

These results indicate that only in patients who later developed LID, but not in the No-LID group, levodopa gradually increased connectivity between putamen and M1 during the 20 min after levodopa intake.

**Figure 3:**
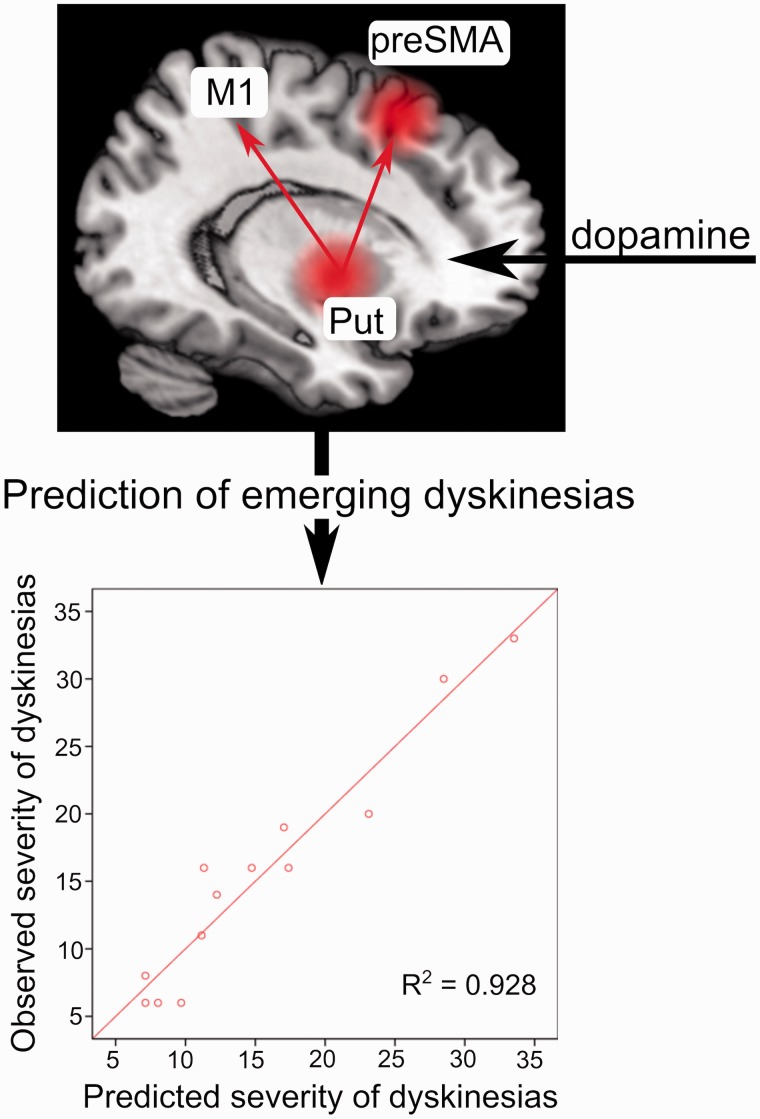
**Dopaminergic modulation of neural networks predicts severity of involuntary movements**. The figure illustrates that mapping of the neural network reorganization after dopamine intake allowed precise predictions about the severity of LID even before they emerged. *Top*: Red spheres indicate changes in activity of preSMA and putamen, which were related to the emergence of LID. Red arrows indicate that feedback connectivity from the putamen to M1 and preSMA significantly improved predictions of LID compared to a regression model, which only contained changes in neural activity. *Bottom*: The panel shows that the hierarchical multiple linear regression model explained almost 93% variance of interindividual differences in LID scores.

In a final step, we applied regression analysis to test whether this dynamic network reorganization following levodopa predicted the individual severity of LID. For each patient of the LID group, we extracted the estimated coupling values reflecting levodopa-induced changes in network connectivity (B-matrix in DCM) based on the best model (Model 9). We then entered these values into a hierarchical multiple linear regression model. The individual connectivity estimates were added to a regression model only containing activity changes (preSMA and putamen, see ‘Patients and methods’ section) to specifically assess the associations between acute levodopa induced changes in neural connectivity and LID severity. Entering coupling values of ‘feedback’ connections from putamen to preSMA (*P*_*corrected*_ = 0.020) and M1 (*P*_*corrected*_ = 0.044), respectively significantly improved the proportion of explained variance in individual LID scores as opposed to a model only containing modulation of neural activity. Modulation of neural connectivity improved predictions of LID severity by ∼22% (R^2^-change = 0.218) resulting in prediction of ∼93% (R^2^ = 0.928, adjusted R^2^ = 0.877) of inter-individual variance in LID scores based on a multiple regression model containing levodopa-induced modulation of neural activity and connectivity. This is illustrated in Fig. 3. In contrast, inclusion of levodopa-induced changes in ‘feedforward’ connectivity from preSMA and M1 to the putamen did not significantly improve the proportion of explained variance (*P*_*corrected*_ = 1). Dopaminergic modulation of striato-cortical connectivity did not correlate with UPDRS-OFF scores or disease duration (all *P*_*uncorrected*_ > 0.1).

## Discussion

In this study, we pharmacologically induced acute changes in effective connectivity with a single dose of the dopamine precursor levodopa and traced the dynamic changes in effective connectivity between the putamen and key cortical motor areas in the pre-dyskinesia period. This novel dynamic functional MRI approach revealed for the first time, an abnormal dopaminergic modulation of striato-cortical connectivity in patients who suffer from LID compared to Parkinson's disease patients without LID. Indeed, the effect of levodopa on neural activity and connectivity accurately predicted the severity of subsequent LID (R^2^ > 0.9).

Reinforcement of specific connections between the cortex and basal ganglia is mediated by neural plasticity, in particular long-term potentiation, in the synapses projecting onto the medium-spiny neurons in the putamen (Calabresi *et al*., 2007). Long-term potentiation is induced when dopamine, which is released from the endings of dopaminergic nigro-striatal projections, and glutamate, which is released by cortico-striatal projections, simultaneously activate the medium-spiny neurons in the putamen (Calabresi *et al*., 2007). During progression of Parkinson's disease, the ability to release dopamine in a controlled manner is severely affected, which leads to an abnormal induction of long term potentiation at the cortico-striatal synapse when patients take their dopaminergic medication (Cenci and Lundblad, 2006). Such a mechanism is thought to cause an aberrant enhancement of striato-cortical connections leading to emergence of LID. This hypothesis is supported by studies in animals demonstrating an aberrant regulation of long-term potentiation in rats with dyskinesias (Picconi *et al*., 2003; Iravani and Jenner, 2011) and studies using transcranial magnetic stimulation showing abnormal plasticity-like effects in the motor cortex of dyskinetic Parkinson's disease patients (Morgante *et al*., 2006; Huang *et al*., 2011).

Our connectivity analysis provides strong *in vivo* support to the notion that excessive striatal responsiveness to levodopa and aberrant striato-cortical connectivity are key mechanisms underlying peak-of-dose LID in patients with Parkinson's disease. We demonstrate that intake of levodopa triggers an abnormal modulation of neural connectivity between putamen and frontal cortical areas in patients with Parkinson's disease with LID compared to patients with Parkinson's disease without LID. Analysis of effective connectivity using DCM demonstrated that in patients who later developed LID, a levodopa-induced rise in dopamine gradually enhanced connectivity between the putamen and M1. This pharmacodynamic modulation of effective connectivity between putamen and M1 was absent in those patients with Parkinson's disease showing a stable clinical response to levodopa without LID.

The connectivity changes induced by a single dose of levodopa in patients with Parkinson's disease with LID displayed several features that deserve comment. First, the abnormal network response in the LID group was expressed during NoGo trials. Therefore, we hypothesize that the increased influence of the putamen to M1 might reflect an (inappropriate) ‘Go’ signal that gradually emerges despite a cue-response mapping rule requiring movement suppression. Second, the aberrant striato-cortical coupling emerged very soon, showing a gradual build-up over 20 min after levodopa intake. In the current study the LID group received a significantly higher dose of levodopa compared to the No-LID group. One might therefore speculate whether the abnormal dopaminergic modulation of striato-cortical connections in the LID group observed in this study might be related to an abnormal sensitization of postsynaptic D1 receptors induced by pulsatile stimulation and abnormally elevated levels of synaptic dopamine during levodopa treatment. Third, the emergence of abnormal striato-cortical connectivity predicted the severity of subsequent LID in a regression model that accounted for changes in regional activity. This predictive value of the observed connectivity pattern suggests a causal link between the levodopa-induced change in striato-cortical connectivity and the manifestation of peak-of-dose LID in our LID patient cohort. Critically, this abnormal expression of striato-cortical connectivity was not confounded by differences in behaviour or the presence of LID. Patients with and without LID showed no differences in task performance. Further, we closely monitored that LID patients had no dyskinesias during data acquisition.

Our findings are in good agreement with electrophysiological studies suggesting that the clinical effect of dopamine in Parkinson's disease is mediated by connections from the basal ganglia to the cortex (Williams *et al*., 2002). Regarding the directionality of connectivity changes, our regression analysis showed that modulation of feedback connections from the putamen to preSMA and M1, but not feedforward connections, strongly predicted severity of day-to-day symptomatic LID. Furthermore, analysis of neural connectivity in this study was based on neural activity recorded during NoGo trials, i.e. trials in which actions (and thus cortico-striatal connectivity) should not be facilitated. Even though we cannot disentangle connections processed via the direct and indirect pathway, respectively, the finding that connectivity changes related to the severity of LID were directed from the putamen to cortical areas indicates that the observed connectivity changes reflect an aberrant reinforcement signal. Such a mechanism would result in abnormally decreased thresholds to generate a movement and thus the emergence of involuntary dyskinesia movements.

In conclusion, we demonstrate for the first time abnormal dopaminergic modulation of striato-cortical connectivity underlying LID in humans. Our findings highlight that dopamine has aberrant effects on the dynamic organization of neural networks when applied in a non-physiological manner in patients with Parkinson's disease. Our pharmacodynamic functional MRI approach has the potential to uncover abnormal striato-cortical connectivity patterns in other brain diseases where symptoms are treated with neuromodulatory drugs. In Parkinson's disease, our approach might also help to identify patients at risk of developing side effects to dopaminergic medication by mapping their individual neural response to dopamine.

## Supplementary Material

Supplementary Table 1

## Abbreviations

DCM: dynamic causal modelling
LID: levodopa-induced dyskinesias
preSMA: pre-supplementary motor area
UPDRS: Unified Parkinson's Disease Rating Scale
