# Supplementary material for: Abnormal dopaminergic modulation of striato-cortical networks underlies levodopa-induced dyskinesias in humans
Source: Brain. 2015 Apr 15;138(6):1658–66. doi: 10.1093/brain/awv096 (PMC4614130; doi:10.1093/brain/awv096)
Supplement: Supplementary Table 1 [file brain_awv096_index.html]

Supplementary Data | Brain

## Supplementary Data

files

- Supplementary Data - pdf file
- Supplementary Data - jpg file
