# Supplementary material for: Abnormal dopaminergic modulation of striato-cortical networks underlies levodopa-induced dyskinesias in humans
Source: Brain. 2015 Apr 15;138(6):1658–66. doi: 10.1093/brain/awv096 (PMC4614130; doi:10.1093/brain/awv096)
Supplement: Supplementary Table 1 [file eea0c7aaae49e4cdfb604e880c36f09e_brain-2014-02163-File005.pdf]

Variable	LID	No-LID	*P* value (uncorrected)
Gender	7f/6m	4f/9m	0.428
Handedness	11r	12r	1
Age (y)	68.9 ± 10.4	67.5 ± 6.5	0.705
Education (y)	14.9 ± 3.6	13.7 ± 3.4	0.406
MMSE	29.2 ± 1	29.6 ± 0.9	0.309
MoCA	28.3 ± 1.4	28.6 ± 1	0.538
BIS	57.4 ± 7.5	55.5 ± 7.6	0.522
Disease Duration (y)	7.5 ± 4.2	6.1 ± 3.3	0.336
LEDD (all medicine)	974.2 ± 415.9	672.3 ± 256.7	0.036*
LEDD (levodopa)	823 ± 396	454 ± 227	0.009*
LEDD (agonists)	162 ± 115	218 ± 135	0.267
Medicine (Agonists)	10	12	0.593
UPDRS-III-OFF	32.5 ± 10.5	32.9 ± 6.8	0.912
UPDRS-III-ON	20 ± 7.5	21.2 ± 5.1	0.630
Δ UPDRS-III	12.5 ± 4.9	11.6 ± 4	0.634
UDysRS (obj.)	15.46 ± 8.7	-	-

Supplementary table 1. Clinical and demographic characteristics of the LID and No-LID group.

Gender and handedness were compared using chi-square tests. Chi-square test was also used to compare whether dopamine agonists were more frequently added to levodopa therapy in LID (n = 10/13) compared to No-LID patients (n = 12/13). Age, education, MMSE, MoCA, BIS, disease duration, LEDD (all dopaminergic medication, levodopa, agonists), UPDRS-OFF, -ON, and –ON vs. OFF

were compared using independent samples t-tests. r, right; y, years. UPDRS-ON was assessed ~ 45 min after intake of 200 mg fast-acting soluble levodopa (i.e. after the MRI session). * indicates significant differences between groups.

	preSMA			right M1			left M1			right putamen			left putamen		
	x	y	z	x	y	z	x	y	z	x	y	z	x	y	z
fMRI	-4	8	58	40	-24	56	-38	-28	52	34	0	4	-28	10	-8
DCM															
mean	-5	12	54	46	-22	58	-44	-27	57	25	-1	3	-23	6	-4
sd	8	4	8	6	8	4	6	6	9	4	4	7	4	11	6

Supplementary table 2. Mean coordinates for the DCM analysis.

"fMRI" shows the group maxima of the respective fMRI contrasts for the regions considered in the DCM (Left: right M1; Right: left M1, NoGo: preSMA, right and left putamen). For each individual participant coordinates were moved to the individual maxima in the respective anatomical region. The mean and standard deviation (sd) of the resulting coordinates are shown.

B-matrix		LID (model 9)	No-LID (model 7)
	preSMA-rPut	-0.07	-0.04
	preSMA-lPut	-0.20	+0.07
	rPut-preSMA	+0.06	+0.11
	lPut-preSMA	+0.02	+0.09
	rM1-rPut	-0.19	-
	lM1-lPut	-0.04	-
	rPut-rM1	-0.31	-
	lPut-lM1	+0.17	-
C-matrix			
Left	rM1	+0.28	+0.37
Right	lM1	+0.27	+0.36
NoGo	preSMA	-0.03	-0.01
	rPut	+0.03	-0.02
	lPut	+0.0004	-0.07
A-matrix			
	rPut-preSMA	-0.04	+0.002
	lPut-preSMA	+0.10	+0.02
	lM1-rM1	+0.20	-0.01
	rPut-rM1	+0.16	+0.08
	lPut-rM1	-0.01	-0.001
	rM1-lM1	+0.03	+0.05
	rPut-lM1	-0.06	-0.01
	lPut-lM1	-0.03	+0.04
	preSMA-rPut	+0.26	+0.18
	rM1-rPut	-0.03	-0.19
	lM1-rPut	-0.62	-0.06
	lPut-rPut	+0.21	+0.09
	preSMA-lPut	+0.06	-0.16
	rM1-lPut	-0.52	+0.04
	lM1-lPut	+0.28	+0.64
	rPut-lPut	+0.15	+0.06

Supplementary table 3: Estimated DCM parameters

The table shows the parameters (B-, C- and A-matrix) for all considered regions and connections separately for the respective best model of the LID group (model 9) and No-LID group (model 7).
