# Supplementary figures and images for: Abnormal dopaminergic modulation of striato-cortical networks underlies levodopa-induced dyskinesias in humans
Source: Brain. 2015 Apr 15;138(6):1658–66. doi: 10.1093/brain/awv096 (PMC4614130; doi:10.1093/brain/awv096)

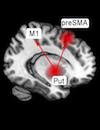

Supplement: Supplementary Table 1 [file e41ed0e9c3db4b6337b563f35e6af3be_brain-2014-02163-File006.jpg]
